# Direct *in vivo* assessment of global and regional mechanoelectric feedback in the intact human heart

**DOI:** 10.1016/j.hrthm.2021.04.026

**Published:** 2021-08

**Authors:** Michele Orini, Peter Taggart, Anish Bhuva, Neil Roberts, Carmelo Di Salvo, Martin Yates, Sveeta Badiani, Stefan Van Duijvenboden, Guy Lloyd, Andrew Smith, Pier D. Lambiase

**Affiliations:** ∗ Electrophysiology Department, Barts Heart Centre at St. Bartholomew's Hospital, London, United Kingdom; † Institute of Cardiovascular Science, University College London, London, United Kingdom

**Keywords:** Arrhythmia, Cardiac strain, Electromechanical coupling, Mechanoelectric feedback, Repolarization

## Abstract

**Background:**

Inhomogeneity of ventricular contraction is associated with sudden cardiac death, but the underlying mechanisms are unclear. Alterations in cardiac contraction impact electrophysiological parameters through mechanoelectric feedback. This has been shown to promote arrhythmias in experimental studies, but its effect in the *in vivo* human heart is unclear.

**Objective:**

The purpose of this study was to quantify the impact of regional myocardial deformation provoked by a sudden increase in ventricular loading (aortic occlusion) on human cardiac electrophysiology.

**Methods:**

In 10 patients undergoing open heart cardiac surgery, left ventricular (LV) afterload was modified by transient aortic occlusion. Simultaneous assessment of whole-heart electrophysiology and LV deformation was performed using an epicardial sock (240 electrodes) and speckle-tracking transesophageal echocardiography. Parameters were matched to 6 American Heart Association LV model segments. The association between changes in regional myocardial segment length and activation-recovery interval (ARI; a conventional surrogate for action potential duration) was studied using mixed-effect models.

**Results:**

Increased ventricular loading reduced longitudinal shortening (*P* = .01) and shortened ARI (*P* = .02), but changes were heterogeneous between cardiac segments. Increased regional longitudinal shortening was associated with ARI shortening (effect size 0.20 [0.01–0.38] ms/%; *P* = .04) and increased local ARI dispersion (effect size –0.13 [–0.23 to –0.03] ms/%; *P* = .04). At the whole organ level, increased mechanical dispersion translated into increased dispersion of repolarization (correlation coefficient r = 0.81; *P* = .01).

**Conclusion:**

Mechanoelectric feedback can establish a potentially proarrhythmic substrate in the human heart and should be considered to advance our understanding and prevention of cardiac arrhythmias.

## Graphical abstract

**Figure:**
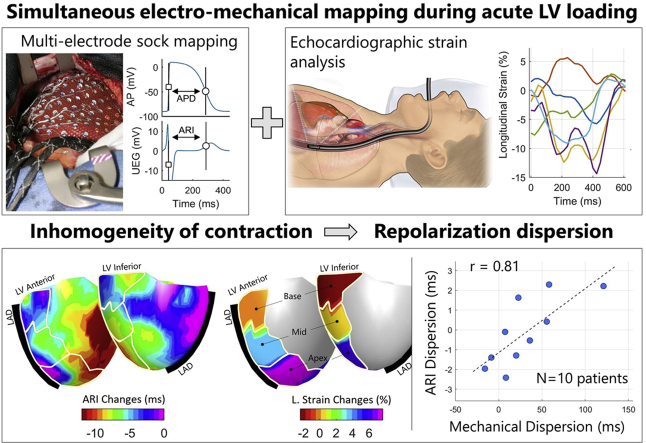

## Introduction

Mechanoelectric feedback (MEF) is an established mechanism whereby myocardial deformation causes changes in cardiac electrophysiological parameters.^1^ Animal, laboratory, and theoretical investigations have demonstrated that abnormal patterns of cardiac deformation can modulate electrical excitation and recovery through MEF, which can be proarrhythmic.^1^, ^2^, ^3^ Indeed, stretch-activated ventricular arrhythmias are well-recognized clinical phenomena described in commotio cordis, mitral valve prolapse, and infarct border zones.^4^, ^5^, ^6^, ^7^, ^8^ Furthermore, echocardiographic parameters of myocardial contractile function measured as strain (relative deformation of myocardial segments) and its spatial dispersion are established risk factors for ventricular arrhythmias in patients with impaired left ventricular (LV) function.^9^, ^10^, ^11^ However, how mechanical perturbations commonly seen in these cardiac patients translate into a proarrhythmic electrophysiological substrate remains unclear. The few studies that have tried to address this knowledge gap have been limited to single-site recordings, which cannot capture mechanical desynchrony and spatial heterogeneity of electrophysiological parameters, which is a primary factor in the establishment of a proarrhythmic substrate.^12^, ^13^, ^14^, ^15^ This is particularly important in view of the characteristically inhomogeneous nature of electrophysiological and mechanical properties of the human heart, which are known to be increased in pathological conditions. We hypothesized that global fluctuations in ventricular loading, common in cardiac conditions such as heart failure, are translated into regionally inhomogeneous changes in mechanical function, which then induce regionally inhomogeneous changes in the electrophysiology by MEF, thereby enhancing dispersion and creating a potentially proarrhythmic substrate.

**Figure 1:**
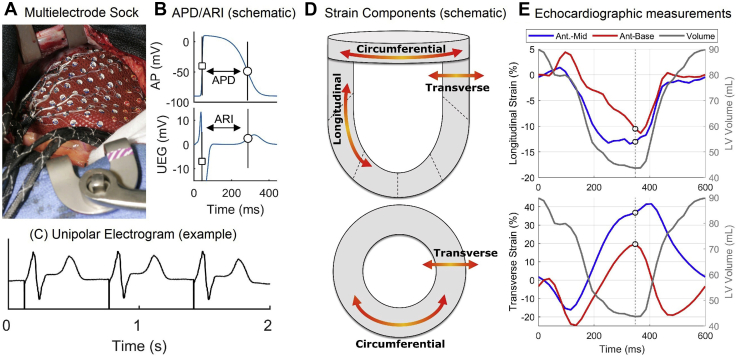
**A:** Epicardial sock placed around a patient’s heart during surgery. (Adapted from Taggart et al.^17^) **B:** Activation-recovery interval (ARI), a surrogate for action potential duration (APD), is measured from the unipolar electrogram (UEG) as the difference between activation *(square)* and repolarization *(circle)* times. **C:** Representative examples of unfiltered UEGs. **D:** Schematic showing longitudinal, transverse, and circumferential components of myocardial deformation. **E:** Fractional change in longitudinal and transverse segment length (strain) are illustrated for 2 cardiac segments (anterior base *[red line]* and anterior–mid myocardium *[blue line]*) along with intraventricular volume *(solid gray line)*. Measurements were taken at end-systolic volume *(dotted vertical line)*. Note that longitudinal strain values are negative, indicating relative shortening. AP = action potential.

In this study, we quantified the impact of regional myocardial deformation due to changes in LV loading on electrical excitation and recovery in the *in vivo* human heart. This was achieved through a unique experimental model that enabled simultaneous quantification of electrophysiology (through high-density, 240-electrode mapping) and cardiac mechanics (through speckle-tracking echocardiography) during manipulation of ventricular loading using the established method of transient aortic occlusion in patients undergoing open heart surgery (Figure 1).^12^

## Methods

A detailed description of the methods is provided in the Supplemental Methods.

### Experimental setting

The study was approved by the local Ethics Committee (reference number 05/Q0502/45) and was conducted in accordance with the Declaration of Helsinki. All patients gave written informed consent. Cardiac mapping and transesophageal echocardiography (TEE) were simultaneously performed in 10 patients (4 women; median 63, interquartile range 60–71 years old) undergoing cardiac surgery incorporating cardiopulmonary bypass (7 coronary artery bypass grafting, 2 aortic valve replacement, 1 both).^16^^,^^17^ A multielectrode heart sock enabling the recording of 240 unipolar electrograms was fit over the epicardium and aligned to the left anterior descending artery using landmarks and electrode labels to enable anatomic segmentation and coregistration with echocardiographic data. Ventricular pacing was established with pacing rate, pulse duration, and amplitude set to ensure consistent capture (20 bpm above sinus rhythm, 1 ms, and twice diastolic threshold, respectively, in most patients). A transient aortic clamp of 4–6 beats was performed to alter ventricular loading. If this induced ectopics, a second clamp was performed after 2 minutes. TEE recordings were taken before, during, and after occlusion in a standard 2-chamber view using an ultrasound machine (iE33, Philips, Netherland) enabling 2-dimensional speckle tracing for myocardial deformation analysis (Figure 1).

### Data analysis

#### Electrophysiological parameters

Activation time (AT) and repolarization time (RT) were estimated from the unipolar electrograms using validated methods, and activation-recovery interval (ARI), an established measure of action potential duration (APD), was calculated as ARI = RT – AT (Figure 1).^18^^,^^19^ Signal processing was performed with bespoke algorithms as in previous studies.^19^^,^^20^ AT, ARI, and RT from each electrode of the heart sock were averaged during aortic occlusion and during 4 beats preceding and following it. Electrodes were matched to 6 segments of the American Heart Association (AHA) LV model for comparison with regional echocardiographic analysis (basal, mid, and apical segments in the anterior and inferior portions of the LV). Mean and standard deviation of ARI across electrodes within each anatomic segment were computed to assess regional ARI and regional ARI dispersion, respectively. The range of regional ARI was computed as a measure of global electrophysiological dispersion. Assessment of regional AT and AT dispersion, as well as global AT dispersion, was conducted in the same way.

#### Echocardiographic parameters

TEE segments were analyzed using commercial software integrating speckle tracking (TomTec Arena 1.4, TomTec Imaging Systems, Unterschleissheim, Germany). Image segmentation was performed semiautomatically by an expert cardiologist blinded to electrophysiological results, according to international consensus.^21^ Deformation parameters were measured from a single beat showing stable waveforms. Myocardial segment length was measured in 6 AHA segments in the basal, mid, and apical segments in the anterior and inferior portions of the LV. Change in myocardial segment length from end-diastole to end-systole as a percent of end-diastolic length (strain) was measured, with a negative value indicating segment shortening and a positive value indicating segment lengthening. For example, a segment of 1 cm that stretches to 1.5 cm or contracts to 0.5 cm would have +50% or –50% strain, respectively. In sensitivity analysis, strain was measured as the fractional change in segment length from end-diastole to its peak value within the cardiac cycle. Global mechanical dispersion was assessed as the standard deviation of time to peak change in myocardial segment length across the 6 anatomic segments.^9^

### Statistical analysis

Continuous variables are presented as median (interquartile range). The Wilcoxon sign-rank test was used to test paired comparisons (before vs during occlusion). Changes due to aortic occlusion were measured in terms of differences between parameters registered during and before occlusion. Correlation was assessed using the Spearman correlation coefficient. Mixed-effect regression models were used to study the association between electrophysiological changes and deformation parameters at the regional level. These models use data structured in a hierarchical way efficiently while reducing problems related to pseudo-replication. Electromechanical interactions across cardiac segments within the same subject were modeled as fixed effects, whereas interpatient variability was considered a random effect.

## Results

**Figure 2:**
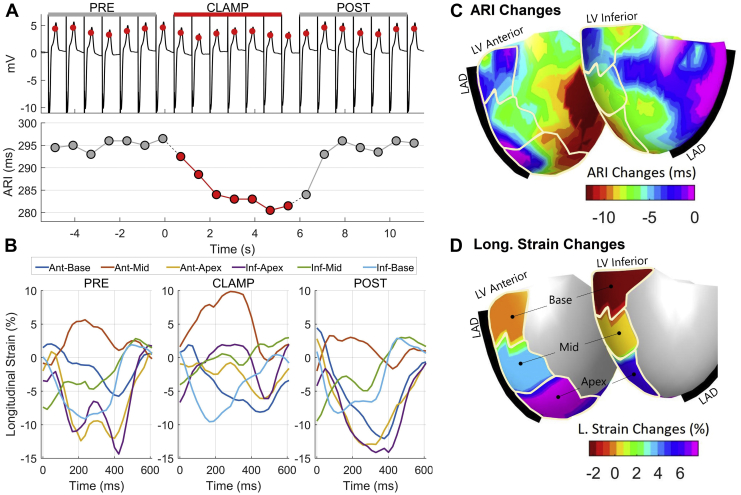
Effect of transient aortic occlusion on electrophysiological and myocardial deformation parameters in a representative patient. **A:** Unipolar electrograms with repolarization time markers (**top)***(red circles*) and activation-recovery interval (ARI) **(bottom)** before, during *(red)*, and after occlusion. **B:** Waveforms representing longitudinal strain in 6 left ventricular (LV) segments of the standardized American Heart Association (AHA) LV model during a single cardiac cycle before, during, and after occlusion. **C:** Changes in ARI during aortic clamp mapped over the heart sock geometry. **D:** Changes in longitudinal strain during aortic clamp mapped over the heart sock geometry. Stylized left anterior descending artery (LAD) and LV AHA segments are shown for orientation.

In 2 patients, ectopic beats were induced during the first aortic clamp but not during the second one. Increased ventricular loading increased LV cavity size and altered myocardial contractility and ventricular repolarization. An example from a representative patient is shown in Figure 2. Both ARI (Figure 2A) and myocardial segment length (strain) (Figure 2B) temporarily changed during increased ventricular loading (aortic occlusion). Our experimental model enabled simultaneous mapping and coregistration of ARI (Figure 2C) and myocardial strain (Figure 2D) changes over 6 LV segments.

### Global effect of increased ventricular loading

Transient aortic occlusion induced significant changes in parameters describing global contractility and electrophysiology (Table 1). These included a significant reduction in LV ejection fraction (median variation –15.6% compared to preocclusion; *P* = .004), global longitudinal (*P* = .01) and circumferential shortening (*P* = .004), and transverse thickening (*P* = .014). These changes indicate a worsening of myocardial function with increased ventricular loading compared to baseline conditions. ARI values changed by up to 15 ms during increased loading, with median ARI across the entire LV decreasing from 255 (228–268) ms before clamp to 252 (227–263) ms during clamp (*P* = .02).

**Table 1:** Global parameters of myocardial deformation and electrophysiology before (Pre) during (Occlusion), and after (Post) transient aortic occlusion

	Pre	Occlusion	Post
Mean LV ARI ms	255 (228–268)	**252 (227–263)∗**	254 (230–269)
SD of LV ARI (ms)	16.0 (13.5–19.5)	16.8 (13.7–18.4)	17.0 (13.7–19.9)
Mean LV AT (ms)	49.6 (43.1–52.5)	49.7 (42.8–52.5)	49.4 (43.1–53.5)
SD of LV AT (ms)	22.2 (17.2–23.5)	22.3 (17.4–23.4)	22.2 (17.1–23.5)
Mean LV RT (ms)	306 (288–315)	**302 (282–311)^†^**	305 (288–316)
SD of LV RT (ms)	21.8 (18.7–29.5)	23.0 (18.4–29.5)	22.7 (18.7–30.5)
EDV (mL)	99.6 (89.5–135.1)	110.0 (72.1–143.8)	109.5 (94.0–131.1)
ESV (mL)	48.8 (43.8–83.3)	57.5 (46.2–92.5)	56.4 (35.2–88.2)
LVEF (%)	44.9 (41.5–51.6)	**37.9 (33.5–46.8)^†^**	47.8 (42.8–59.7)
GLS (%)	–9.69 (–11.30 to 6.55)	**–6.77 (–9.76 to 4.77)^†^**	–10.74 (–18.24 to 5.92)
GCS (%)	–19.9 (–21.5 to 16.6)	**–14.0 (–18.1 to 13.1)^†^**	–17.5 (–25.7 to 13.3)
GRS (%)	23.7 (14.4–27.3)	**18.2 (9.8–19.9)∗**	18.4 (9.7–30.6)

Values are given as median (1st–3rd quartile) across patients (n = 10). For each patient, mean and standard deviation (SD) of activation time (AT), repolarization time (RT), and activation recovery interval (ARI = RT-AT) were measured across all electrodes covering the left ventricle (LV). Values statistically different from preocclusion are shown in bold (∗*P* <.05; **^†^***P* <.01).

EDV = end-diastolic volume; ESV = end-systolic volume; GCS = global circumferential strain; GLS = global longitudinal strain; GRS = global radial strain; LVEF = left ventricular ejection fraction.

### Correlation between regional cardiac deformation and electrophysiology

**Figure 3:**
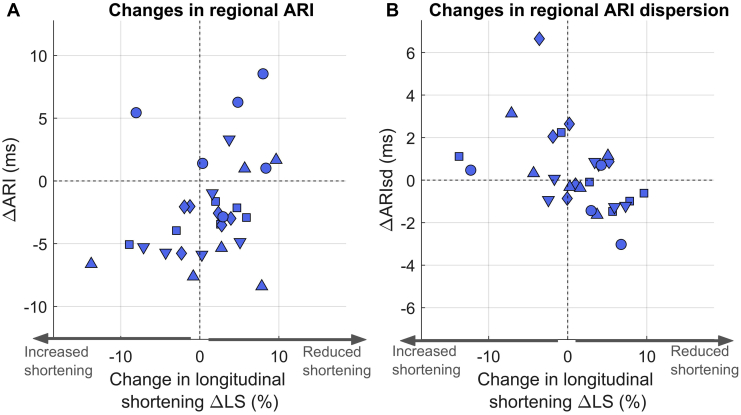
Correlation between myocardial deformation and repolarization secondary to increase in ventricular loading for 5 patients. Each symbol represents a cardiac segment, and each marker type represents a patient (6 cardiac segments per patient). Changes in regional longitudinal shortening (ΔLS) correlated with changes in regional activation-recovery interval (ΔARI) and inversely correlated with changes in regional ARI dispersion (ΔARIsd).

Regional changes in myocardial deformation and repolarization were heterogeneous. The ARI decrease was not uniformly distributed across the LV. Although median ARI decrease across patients was 4 (3–5) ms, some cardiac sites showed substantial reductions in ARI, with maximal ARI decreases of 15 (13–20) ms. At the same time, 18.3% of all cardiac segments showed an increase in ARI. Similarly, although longitudinal segment shortening was decreased during occlusion in most segments, 36.7% of all segments showed an increase in longitudinal segment shortening. At the regional level, changes in longitudinal segment shortening significantly correlated to regional ARI and ARI dispersion (ie, spatial heterogeneity of repolarization). Mixed-effect models identified a significant positive association between changes in ARI and changes in longitudinal strain (effect size 0.20 [0.01/0.38] ms/%; *P* = .04). This indicates regional ARI reduction in segments with increased longitudinal shortening and regional ARI increase in segments with reduced longitudinal shortening (Figure 3A). Changes in longitudinal shortening were also significantly associated with changes in regional ARI dispersion (effect size –0.13 [–0.23/–0.03] ms/%; *P* = .01). This indicates that spatial heterogeneity of ARI increased in segments with greater longitudinal shortening (Figure 3B).

Regional changes in longitudinal segment length at baseline (ie, before transient aortic clamping) were associated with regional ARI (*P* = .067) and regional ARI dispersion (*P* = .024) (Supplemental Table 1). No significant associations were identified between regional myocardial deformation parameters and regional AT, whereas changes in total repolarization (ie, RT=AT + ARI) showed associations with myocardial deformation parameters akin to those described for ARI (Supplemental Table 1).

**Figure 4:**
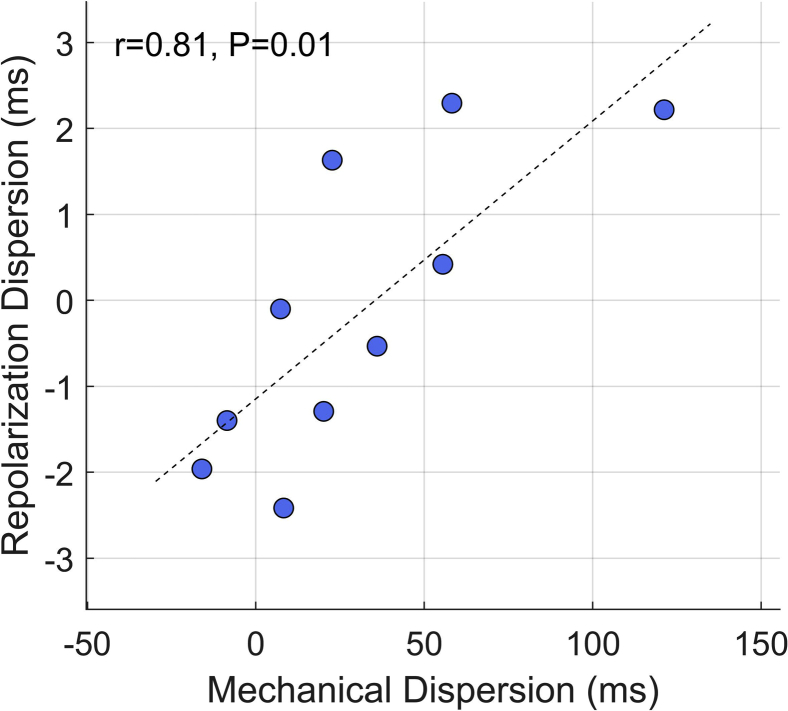
Translation of global mechanical dispersion into global electrophysiological dispersion. During increase in ventricular loading, changes in mechanical dispersion, measured as the standard deviation of time to peak longitudinal shortening (strain), correlated with global activation-recovery interval (ARI) dispersion, measured as the standard deviation of regional ARI. Each symbol represents a patient (n = 10). The correlation coefficient (r) is reported for each scatterplot.

At the patient level, changes in global dispersion of ARI (ie, ARI heterogeneity between segments) strongly correlated to mechanical dispersion (r = 0.81; *P* = .01) (Figure 4). This indicates that increased mechanical desynchrony translated into increased dispersion of repolarization. A moderate but nonsignificant correlation was also found between global dispersion of ARI and the standard deviation of longitudinal strain between segments (r = 0.50; *P* = .14).

Sensitivity analysis conducted using peak strain values showed similar results (Supplemental Table 2). Regional longitudinal strain remained significantly implicated in the changes observed in regional ARI and regional ARI dispersion, and the effect sizes were similar.

### Activation-repolarization interactions

Experimental models in animals have shown that the normal inverse relationship between AT and RT is modulated by alterations in ventricular loading.^22^ In the current study, across electrodes covering the LV, maximum ARI shortening was significantly greater for sites activating late (ie, after median AT) than for sites activating early (ie, before median AT) at 14.2 (11.5–20.3) ms vs 12.4 (9.3–13.7) ms (*P* = .01_, respectively. Median value of ARI shortening for late-activated LV sites was numerically greater than for early-activated sites (4.2 [2.5–6.04] ms vs 3.3 [2.7–4.3] ms), but the difference was not statistically significant (*P* = .19).

## Discussion

This study provides quantitative and simultaneous assessment of the effect of myocardial deformation on ventricular electrophysiology during a sudden change in ventricular loading. The transient aortic occlusion utilized in this model to alter ventricular loading significantly modified contractility (longitudinal, transverse, and circumferential myocardial strain) and electrophysiology. The main findings of the study are that (1) global changes in ventricular loading produce regional changes in mechanical function and electrophysiology, which were inhomogeneous; (2) changes in regional longitudinal shortening were directly related to changes of both regional repolarization and regional repolarization dispersion; and (3) global mechanical dispersion, a measurement of desynchrony in contraction, increased global dispersion of repolarization. These results are consistent with the hypothesis that increase in ventricular loading produces inhomogeneous changes in both mechanical function and electrophysiology between different regions as well locally within regions through the intermediary of MEF. This is especially important in the presence of underlying heart disease, such as ischemic heart disease and cardiomyopathy, which are characteristically associated with the development of scar and fibrosis, both of which are patchy and promote contraction inhomogeneity. These results have important implications in the understanding of fundamental mechanisms underlying potentially life-threating ventricular arrhythmias in the human heart because they implicate impaired myocardial contraction and desynchrony in the modulation of regional and global spatial dispersion of repolarization, which are well-established proarrhythmic factors.^23^, ^24^, ^25^ This could at least partially explain the association between mechanical dispersion and ventricular arrhythmia or sudden cardiac death, established from clinical studies.^9^, ^10^, ^11^

The analysis of regional mechanoelectric interactions shows that changes in the mechanical properties of a given cardiac segment not only directly impact on the electrophysiology of the same segment (Figure 3) but also may affect the electrophysiology of spatially distinct but coupled segments as suggested by laboratory preparations.^26^ This could explain why there was no significant association between time to peak change in myocardial segment length and repolarization at the regional level (ie, on the same segment), but there was a significant correlation between global mechanical dispersion and global dispersion of repolarization (Figure 4).

A previous study using a similar model of increased afterload in patients found a reduction in APD that correlated with peak systolic pressure.^12^ However, regional MEF interactions were not assessed due to lack of echocardiographic data and simultaneous multisite cardiac mapping. The overall mean reduction of ARI during increased loading in the present study using multisite recordings is consistent with the mean reduction in APD in the previous study using single-site recordings. However, it was not appreciated in the earlier work that a significant number of areas in the heart may show an opposite response during loading (ie, increase in ARI during loading) as observed in 18.3% of sites in the present study. Our results indicate that global parameters alone may not be sufficient to characterize mechanoelectric coupling and that physiological behavior may be masked by global averaging and suggest that although single-site recordings provide valuable information, they need to be complemented with multisite information.

### MEF mechanisms

Changes in the mechanical environment of the myocardial cell influence APD by MEF pathways involving stretch-activated channels, calcium cycling, and chemical signaling.^27^, ^28^, ^29^, ^30^, ^31^ The effect of each of these mechanisms on ventricular repolarization is strongly dependent on the nature and timing of the mechanical perturbation. Although the temporal resolution of echocardiography did not allow accurate assessment of the effect of the temporal pattern of myocardial deformation on repolarization changes, we found greater peak ARI reduction in late-activated LV segments compared to early-activated segments.

Although experimental work on cells, tissues, and *in silico* has identified a range of cellular mechanisms and electrophysiological responses to specific alterations in ventricular loading, extrapolation to the *in vivo* human heart is challenging in view of the 3-dimensional complexity of the stress/strain relationships during the cardiac cycle and species differences in cardiomyocyte electrophysiology and species-dependent mechanisms underlying arrhythmias.^32^ In this study, we incorporated a model of increased afterload, as increased afterload is commonly encountered clinically in pathological conditions on both global and regional scales. We observed a predominant shortening of ventricular APD, with some regions showing lengthening occurring immediately following the abrupt onset of increased afterload. Possible mechanisms include stretch-activated nonspecific cation channels (SACs).^30^ SACs have a reversal potential at approximately –30 mV, such that SACs activated at membrane potentials positive to the reversal potential (ie, during the plateau phase) shorten APD and SACs activated at more negative potential (ie, during the later repolarization phase) prolong APD.^28^^,^^30^ Another possible mechanism would be the effect of increased afterload on calcium cycling. Myocardial shortening decreases the affinity of Ca^2+^ for troponin C.^3^ Free sarcoplasmic calcium during the late phase of the action potential is higher in shortened than nonshortened myocardial segments, which would be expected to prolong the action potential by Na/Ca exchange. Therefore, the more isometric contraction associated with the aortic cross-clamping model would be expected to promote the opposite effect, that is, APD shortening.^3^^,^^27^^,^^28^^,^^31^ Recent work has shown that altered mechanical loading may induce rapid local Ca^2+^ release that is not reflected in global Ca^2+^ but confined to localized regions such as the narrow dyadic space between ryanodine receptors and the L-type Ca^2+^ channels.^3^^,^^33^, ^34^, ^35^ This microdomain microsensitivity includes reactive oxygen species (ROS) and nitric oxide (NO) pathway signaling. Although NO signaling tends to operate on a longer time scale, it is possible that the rapid effect of X-ROS signaling in enhancing the efficiency of calcium-induced calcium release may play a role and contribute to arrhythmogenesis by inducing early and delayed afterdepolarizations through Na/Ca exchange.

### Clinical implications

Stretch-activated ventricular arrhythmias are a well-recognized phenomenon in clinical practice, including ventricular fibrillation triggered by commotio cordis. Recently, the role of stretch-activated premature ventricular contractions has become recognized as a mechanism of triggering ventricular fibrillation in mitral valve prolapse.^8^ Dispersion of repolarization is linked to heterogeneities in mechanical dysfunction promoting mechanoelectric differences in ion channelopathies such as long QT syndrome. However, the fact that myocardial infarction is much more common implicates the proarrhythmic effects of stretch in the infarct border zone or dyssynchronous ventricle of left bundle branch block as a proarrhythmic mechanism more widely in the population.

Mechanisms of arrhythmogenesis in the infarct border zone operate on a number of levels, including activation of stretch-activated channels that change APD, slow conduction velocity, and increased dispersion of repolarization on the physiological level, to changes in expression of mechanically modulated ion channels and alterations in connexin phosphorylation at the molecular level through to structural remodeling of tissue architecture, composition, and innervation.^4^, ^5^, ^6^, ^7^ These processes conspire to further promote arrhythmogenicity in the infarct border zone in response to stretch. Variability in the site and degree of stretch in this region can create dispersion of repolarization to enable the initiation of ventricular tachycardia. This region is the target of catheter ablation, which usually focuses on the electrophysiological markers of structural disease and conduction slowing as opposed to regions of increased strain influencing repolarization.

Mechanical deformation parameters are also becoming recognized predictors of arrhythmic events in structural heart disease.^9^, ^10^, ^11^ These could potentially be improved by integration with markers of repolarization dispersion at sites of abnormal strain/increased mechanical dispersion.

### Study limitations

The number of patients included in the study was limited by the inherent difficulty of conducting experimental studies, including electrophysiological and speckle-tracking recordings in the cardiac operating suite. However, the statistical methods utilized make efficient use of the data and detected significant associations. Coregistration of regional strain and electrophysiological data was performed using anatomic landmarks and labels on the heart sock, but remaining imprecision may have affected the results, possibly reducing the significance of some associations. Measurement of electrophysiological and strain parameters may be challenging in some cases. However, to ensure accuracy and robustness, analyses were performed independently utilizing validated software, automatic exclusion of outliers was performed using predetermined criteria, and sensitivity analysis was conducted to check for consistency. We cannot exclude the possibility that placement of the sock around the heart may influence the electrophysiology. However, the sock fits gently around the heart, and if any mechanically induced repolarization changes were induced by its placement, we would expect them to be trivial.

## Conclusion

This study developed a unique *in vivo* human experimental model of acutely increased ventricular loading by combining multisite electrophysiological mapping with simultaneous transesophageal ultrasound and an established aortic cross-clamping protocol in patients undergoing cardiac surgery. The results show that global ventricular loading conditions induce regional differences in myocardial shortening. These changes in myocardial shortening are associated with changes in electrophysiology, most probably by MEF whereby increased mechanical dispersion results in greater dispersion of repolarization. This suggests that MEF can contribute to the establishment of proarrhythmic substrates in patients and may provide a mechanistic explanation for the association between myocardial strain parameters and sudden cardiac death reported in clinical studies.
