# Supplementary material for: Direct in vivo assessment of global and regional mechanoelectric feedback in the intact human heart
Source: Heart Rhythm. 2021 Aug;18(8):1406–13. doi: 10.1016/j.hrthm.2021.04.026 (PMC8353585; doi:10.1016/j.hrthm.2021.04.026)
Supplement: Supplemental Table [file mmc1.docx]

**Direct in-vivo assessment of global and regional mechano-electric feedback in the intact human heart**

Michele Orini *et al*

Contents

[Detailed Methods 1](#_Toc65146940)

[Experimental setting 1](#_Toc65146941)

[Data Analysis 2](#_Toc65146942)

[Electrophysiological parameters 2](#_Toc65146943)

[Echocardiographic parameters 2](#_Toc65146944)

[Statistical analysis 2](#_Toc65146945)

[Supplementary Table 1 4](#_Toc65146946)

[Supplementary Table 2 5](#_Toc65146947)

[References 6](#_Toc65146948)

# Detailed Methods

## Experimental setting

Cardiac mapping and transoesophageal echocardiography (TOE) were simultaneously performed in patients undergoing cardiac surgery incorporating cardiopulmonary bypass ^1,2^. Cardiopulmonary bypass was temporarily commenced to allow the surgeon to fit a multi-electrode heart sock enabling the recording of 240 unipolar electrograms over the epicardium of both ventricles. The heart sock was aligned to the left anterior descending artery using sock landmarks and electrode labels to ensure anatomical segmentation and co-registration with echocardiographic data. The heart was then refilled, and bypass discontinued in order to study a normally beating heart. Once baseline haemodynamic were restored, pacing impulses were delivered by a cardiac stimulator (Micropace EP Inc.), which was connected to the acquisition system composed of two amplifiers (Clearsign Lab-Sysyem Pro, Bard Electrophysiology). Ventricular pacing was established with pacing rate and pulses’ duration and amplitude set to ensure consistent capture. In most patients, these were set at 20 bpm above sinus rhythm, 1 ms and twice the diastolic threshold, respectively. If ventricular capture was not consistent, the pulses amplitude and rate were adjusted. Duration and amplitude of the pacing pulses were set to ensure consistent capture (1 ms and twice the diastolic threshold, respectively). Unipolar electrograms were recorded with a sampling rate of 2 KHz, and a bandwidth between 0.01 – 500 Hz. The rib retractor was used as the reference electrode. A transient aortic clamp of 4-6 beats was performed to alter ventricular loading. The extent of aortic occlusion (partial or total) was based upon the clinical condition of the patient at the time of surgery. TOE recordings were taken before, during and after occlusion in a standard 2 chamber view using a Philips iE33 ultrasound machine enabling 2D speckle tracing for myocardial deformation analysis (Figure 1).

## Data Analysis

### Electrophysiological parameters

Activation (AT) and repolarization (RT) times were estimated from the unipolar electrograms using validated methods as the time from the pacing stimulus to the minimum of the first derivative during depolarization phase, min(dV/dt), and as the time from the pacing stimulus to the maximum of the first derivative during the repolarization phase, max(dV/dt), respectively (Figure 1). Activation recovery interval (ARI), an established measure of action potential duration (APD), was calculated as ARI = RT – AT ^3–5^. Signal processing was performed with bespoke algorithms as in previous studies ^5–7^. Signals were filtered between 0.5 – 20 Hz for RT measurements and between 0.5 – 45 Hz for AT measurements. AT, ARI and RT from each electrode of the heart-sock were averaged during aortic occlusion and during 4 beats preceding and following it. Electrodes over the LV were grouped into 6 anatomical segments from the standardized AHA LV model for comparison with regional echocardiographic analysis (basal, mid and apical segments in the anterior and inferior portion of the LV). Mean and standard deviation of ARI across electrodes within each anatomical segment were computed to assess regional ARI and regional ARI dispersion, respectively. The range of regional ARI was computed as a measure of global electrophysiological dispersion. Electrophysiological changes induced by aortic clamp were quantified as differences between values measured during the clamp and values measured before the clamp. Assessment of regional AT and AT dispersion, as well as global AT dispersion, was conducted in the same way.

### Echocardiographic parameters

TOE segments were exported and analysed off-line using commercial software (TomTec Arena 1.4, TomTec Imaging Systems, Unterschleissheim, Germany). Image segmentation was performed semi-automatically by an expert cardiologist blinded to electrophysiological results, according to international consensus ^8^. Deformation parameters before and during clamp were measured from a single beat showing stable waveforms. Myocardial strain and strain rate along the longitudinal and transverse (or radial) directions were measured in 6 segments of the standardized AHA LV model (basal, mid and apical segments in the anterior and inferior portion of the LV) using the commercial speckle-tracking software. No segmental measurements were available for circumferential strain. Strain was calculated from end-diastole to end-systole for primary analysis, while maximum/minimum values within the cardiac cycle were used for sensitivity analysis. Parameters along the longitudinal direction were calculated by the software at endocardial, mid-myocardial and epicardial level. These were averaged to reduce the number of parameters and increase robustness. Global strain and strain rate were automatically measured by the software using averaged waveforms. Global mechanical dispersion was assessed as the standard deviation of time-to-peak longitudinal strain across the 6 anatomical segments ^9^. Segmental parameters outside the range $[q_{1}-2\times\left( q_{3}-q_{1} \right)]-{[q}_{3}+2\times(q_{3}-q_{1})]$, where $q_{1}$and $q_{3}$ are first and third quartiles of the distribution across all segments and patients, were considered as outliers and excluded from the analysis. This led to the exclusion of 17 segmental values (2.3 % of total values) across all parameters measured before and during occlusion and their difference.

## Statistical analysis

Continuous variables are presented as median/interquartile range. The Wilcoxon sign-rank test was used to test paired comparisons (before vs during occlusion) with statistical significance set at P<0.05. Changes due to aortic occlusion were measured in terms of differences between parameters registered during and before occlusion. The correlation between changes in myocardial deformation and electrophysiological parameters was assessed using the Spearman correlation coefficient. A correlation coefficient was considered significantly different from zero if its P-value was <0.05. Mixed-effect regression models were used to study the association between electrophysiological changes in ARI and AT and deformation parameters at the regional level. These models use data structured in a hierarchical way efficiently while reducing problems related to pseudo-replication. The following electrophysiological parameters were included as dependent variables: changes in regional ARI and regional ARI dispersion. The following deformation parameters were included as independent variables: Changes in longitudinal and transverse strain and strain rate during and before aortic clamp. To understand the effect on electrical vulnerability according to underlying myocardial function, longitudinal and transverse strain and strain rate before clamp were included. Electro-mechanical interactions across cardiac segments within the same subject were modelled as fixed effects, whereas inter-patient variability was considered as a random effect. Interactions between global dispersion of mechanical and electrophysiological parameters were assessed by means of correlation coefficients. Statistical analysis was performed in Matlab R2019b (MathWorks).

# Supplementary Table 1

Regional deformation parameters at end of diastolic volume (primary analysis)

|  | **ΔARIm** | | **ΔATm** | | **ΔRTm** | |
| --- | --- | --- | --- | --- | --- | --- |
| Echo | Coefficient | P | Coefficient | P | Coefficient | P |
| **ΔLSR** | -1.94 (-4.72/0.84) | 0.17 | -0.40 (-0.94/0.14) | 0.14 | -2.34 (-5.19/0.50) | 0.10 |
| **ΔLS** | 0.20 (0.01/0.38) | 0.04 | -0.00 (-0.04/0.04) | 0.98 | 0.20 (0.01/0.38) | 0.04 |
| **ΔTSR** | 0.27 (-0.82/1.37) | 0.62 | 0.03 (-0.21/0.28) | 0.79 | 0.29 (-0.81/1.39) | 0.60 |
| **ΔTS** | -0.06 (-0.11/-0.00) | 0.04 | 0.01 (-0.01/0.02) | 0.33 | -0.05 (-0.11/0.00) | 0.07 |
| **PreLSR** | 1.61 (-1.15/4.37) | 0.25 | 0.52 (-0.01/1.04) | 0.06 | 2.13 (-0.69/4.96) | 0.14 |
| **PreLS** | -0.10 (-0.20/0.01) | 0.07 | -0.01 (-0.03/0.02) | 0.52 | -0.11 (-0.21/-0.00) | 0.04 |
| **PreTSR** | 0.11 (-0.82/1.05) | 0.81 | -0.05 (-0.26/0.16) | 0.63 | 0.07 (-0.87/1.01) | 0.88 |
| **PreTS** | 0.02 (-0.03/0.08) | 0.41 | -0.01 (-0.02/0.01) | 0.31 | 0.02 (-0.04/0.08) | 0.51 |
|  | **ΔARIsd** | | **ΔATsd** | | **ΔRTsd** | |
| Echo | Coefficient | P | Coefficient | P | Coefficient | P |
| **ΔLSR** | 0.52 (-1.04/2.09) | 0.50 | -0.22 (-0.75/0.32) | 0.42 | 1.68 (-0.41/3.76) | 0.11 |
| **ΔLS** | -0.13 (-0.23/-0.03) | 0.01 | -0.00 (-0.04/0.03) | 0.92 | -0.07 (-0.20/0.06) | 0.28 |
| **ΔTSR** | 0.05 (-0.53/0.63) | 0.87 | 0.06 (-0.14/0.25) | 0.57 | 0.29 (-0.49/1.07) | 0.46 |
| **ΔTS** | 0.01 (-0.02/0.04) | 0.69 | -0.01 (-0.02/0.01) | 0.32 | -0.04 (-0.08/-0.00) | 0.05 |
| **PreLSR** | 0.18 (-1.36/1.72) | 0.82 | 0.13 (-0.39/0.65) | 0.62 | -0.55 (-2.64/1.53) | 0.60 |
| **PreLS** | 0.06 (0.01/0.11) | 0.02 | -0.00 (-0.02/0.01) | 0.73 | 0.02 (-0.05/0.10) | 0.50 |
| **PreTSR** | -0.12 (-0.60/0.35) | 0.61 | 0.09 (-0.07/0.25) | 0.25 | -0.03 (-0.68/0.62) | 0.93 |
| **PreTS** | 0.00 (-0.03/0.03) | 0.97 | 0.01 (-0.00/0.02) | 0.24 | 0.03 (-0.01/0.07) | 0.17 |

**Supplementary Table 1:** Effect of mechanical deformation on repolarization and activation times assessed at the cardiac segment level. Regional deformation parameters are estimated at end diastolic volume (primary analysis). Top: Mixed-effect models were implemented to explain the association between changes of regional ARI (ΔARIm), AT (ΔATm) and (ΔRTm) with regional deformation parameters. Bottom: Mixed-effect models were implemented to explain the association between changes of regional heterogeneity in ARI (ΔARIsd), AT (ΔATsd) and (ΔRTsd) with regional deformation parameters. The estimated effect size (confidence interval) is reported alongside with its P-value. Significant (P<0.05) and borderline nonsignificant (0.05<P<0.10) interactions are highlighted in red and yellow, respectively. Δ: Difference of values measured during – before clamp. Pre: Values measured before clamp. LS and LSR: Longitudinal strain and strain rate. TS and TSR: Transverse strain and strain rate.

# Supplementary Table 2

Regional deformation parameters at their peak values (sensitivity analysis)

|  | **ΔARIm** | | **ΔATm** | | **ΔRTm** | |
| --- | --- | --- | --- | --- | --- | --- |
| Echo | Coefficient | P | Coefficient | P | Coefficient | P |
| **ΔLSR** | -1.56 (-4.78/1.66) | 0.34 | -0.01 (-0.65/0.64) | 0.98 | -1.64 (-4.97/1.69) | 0.33 |
| **ΔLS** | 0.21 (-0.04/0.46) | 0.10 | -0.02 (-0.08/0.03) | 0.38 | 0.18 (-0.07/0.44) | 0.15 |
| **ΔTSR** | 0.12 (-1.35/1.58) | 0.88 | -0.03 (-0.35/0.30) | 0.87 | 0.09 (-1.37/1.56) | 0.90 |
| **ΔTS** | -0.05 (-0.11/0.01) | 0.07 | 0.01 (-0.01/0.02) | 0.35 | -0.05 (-0.11/0.01) | 0.11 |
| **PreLSR** | -0.33 (-3.23/2.56) | 0.82 | 0.41 (-0.24/1.06) | 0.22 | 0.05 (-2.83/2.93) | 0.97 |
| **PreLS** | -0.14 (-0.27/-0.00) | 0.05 | -0.00 (-0.04/0.03) | 0.81 | -0.14 (-0.28/-0.01) | 0.04 |
| **PreTSR** | -0.92 (-2.00/0.17) | 0.10 | -0.09 (-0.33/0.16) | 0.48 | -0.97 (-2.05/0.11) | 0.08 |
| **PreTS** | 0.00 (-0.06/0.06) | 0.98 | -0.01 (-0.02/0.01) | 0.32 | -0.00 (-0.07/0.06) | 0.87 |
|  | **ΔARIsd** | | **ΔATsd** | | **ΔRTsd** | |
| Echo | Coefficient | P | Coefficient | P | Coefficient | P |
| **ΔLSR** | -1.34 (-3.09/0.40) | 0.13 | -0.14 (-0.74/0.47) | 0.66 | -1.65 (-3.91/0.60) | 0.15 |
| **ΔLS** | -0.17 (-0.29/-0.04) | 0.01 | -0.01 (-0.05/0.04) | 0.74 | -0.11 (-0.27/0.06) | 0.21 |
| **ΔTSR** | 0.17 (-0.61/0.94) | 0.67 | -0.13 (-0.39/0.13) | 0.34 | -0.51 (-1.55/0.54) | 0.33 |
| **ΔTS** | 0.00 (-0.03/0.03) | 0.90 | -0.01 (-0.02/0.00) | 0.21 | -0.04 (-0.08/0.00) | 0.07 |
| **PreLSR** | 1.16 (-0.24/2.56) | 0.10 | -0.23 (-0.72/0.25) | 0.34 | 1.75 (-0.04/3.54) | 0.06 |
| **PreLS** | 0.07 (0.00/0.13) | 0.04 | -0.01 (-0.03/0.02) | 0.57 | 0.03 (-0.06/0.12) | 0.53 |
| **PreTSR** | 0.18 (-0.33/0.69) | 0.49 | 0.06 (-0.12/0.23) | 0.52 | -0.07 (-0.77/0.62) | 0.84 |
| **PreTS** | 0.01 (-0.02/0.04) | 0.65 | 0.01 (-0.00/0.02) | 0.15 | 0.01 (-0.03/0.05) | 0.62 |

**Supplementary Table 2:** Effect of mechanical deformation on repolarization and activation times assessed at the cardiac segment level. Regional deformation parameters are estimated at their peak value (sensitivity analysis). Top: Mixed-effect models were implemented to explain the association between changes of regional ARI (ΔARIm), AT (ΔATm) and (ΔRTm) with regional deformation parameters. Bottom: Mixed-effect models were implemented to explain the association between changes of regional heterogeneity in ARI (ΔARIsd), AT (ΔATsd) and (ΔRTsd) with regional deformation parameters. The estimated effect size (confidence interval) is reported alongside with its P-value. Significant (P<0.05) and borderline nonsignificant (0.05<P<0.10) interactions are highlighted in red and yellow, respectively. Δ: Difference of values measured during – before clamp. Pre: Values measured before clamp. LS and LSR: Longitudinal strain and strain rate. TS and TSR: Transverse strain and strain rate.

# References

1. Orini M, Taggart P, Srinivasan N, Hayward M, Lambiase PDPD: Interactions between activation and repolarization restitution properties in the intact human heart: In-vivo whole-heart data and mathematical description. PLoS One 2016; 11:e0161765.

2. Taggart P, Orini M, Hanson B, et al.: Developing a novel comprehensive framework for the investigation of cellular and whole heart electrophysiology in the in situ human heart: Historical perspectives, current progress and future prospects. Prog Biophys Mol Biol 2014; 115:252–260.

3. Coronel R, de Bakker JMT, Wilms-Schopman FJG, et al.: Monophasic action potentials and activation recovery intervals as measures of ventricular action potential duration: Experimental evidence to resolve some controversies. Heart Rhythm 2006; 3:1043–1050.

4. Orini M, Srinivasan N, Graham AJ, Taggart P, Lambiase PD: Further Evidence on How to Measure Local Repolarization Time Using Intracardiac Unipolar Electrograms in the Intact Human Heart. Circ Arrhythmia Electrophysiol 2019; 12.

5. Orini M, Taggart P, Lambiase PD: In vivo human sock-mapping validation of a simple model that explains unipolar electrogram morphology in relation to conduction-repolarization dynamics. J Cardiovasc Electrophysiol 2018; 29:990–997.

6. Martin CA, Orini M, Srinivasan NT, et al.: Assessment of a conduction-repolarisation metric to predict Arrhythmogenesis in right ventricular disorders. Int J Cardiol 2018; 271:75–80.

7. Orini M, Graham AJ, Srinivasan NT, et al.: Evaluation of the reentry vulnerability index to predict ventricular tachycardia circuits using high-density contact mapping. Heart Rhythm 2020; 17:576–583.

8. Voigt JU, Pedrizzetti G, Lysyansky P, et al.: Definitions for a common standard for 2D speckle tracking echocardiography: Consensus document of the EACVI/ASE/industry task force to standardize deformation imaging. J Am Soc Echocardiogr Mosby Inc., 2015; 28:183–193.

9. Haugaa KH, Smedsrud MK, Steen T, et al.: Mechanical Dispersion Assessed by Myocardial Strain in Patients After Myocardial Infarction for Risk Prediction of Ventricular Arrhythmia. JACC Cardiovasc Imaging Elsevier Inc., 2010; 3:247–256.
